# A phase 1/2 trial to evaluate the pharmacokinetics, safety, and efficacy of NI-03 in patients with chronic pancreatitis: study protocol for a randomized controlled trial on the assessment of camostat treatment in chronic pancreatitis (TACTIC)

**DOI:** 10.1186/s13063-019-3606-y

**Published:** 2019-08-14

**Authors:** Mitchell L. Ramsey, Janet Nuttall, Phil A. Hart, on behalf of the TACTIC Investigative Team

**Affiliations:** 1 Division of Gastroenterology, Hepatology, and Nutrition, The Ohio State University Wexner Medical Center, 410 West Tenth Avenue, Columbus, OH 43210 USA; 2 Kangen Pharmaceuticals, America LLC, Kansas City, Kansas USA

**Keywords:** Pancreatic diseases, Abdominal pain, Serine protease inhibitor, Camostat

## Abstract

**Background:**

Chronic pancreatitis (CP) is a progressive, fibro-inflammatory disease characterized by enzymatic autoactivation and subsequent fibrotic replacement of acinar cells. A significant proportion of patients develop pain, which may be due to many causes, including perineural inflammation, altered central processing of pain signals, parenchymal structural changes, and ductal obstruction. Currently there are no approved medical treatment options for CP-associated pain. NI-03 (camostat mesilate) is an orally administered serine protease inhibitor that reduces pancreatic enzyme activity and has been widely used for the treatment of CP-associated pain in Japan. The current study will assess the safety and efficacy of NI-03 for reduction of CP-associated pain in the USA.

**Methods:**

The current study consists of two phases. First, a phase I study will be performed to establish the pharmacokinetics and safety profile over a 1-week period following a single dose (100, 200, or 300 mg). Subsequently, a phase II study will be performed consisting of a double-blind, randomized, controlled trial (RCT). This RCT will evaluate the efficacy of each of the three doses of NI-03 given three times daily compared to placebo over 28 days. A 7-day, single-blind, run-in period will precede the double-blind phase to assess baseline pain characteristics. The primary efficacy outcome is the average of worst daily pain scores (numeric rating scale of 0–10) over the terminal 7 days of the study period compared to baseline. Secondary efficacy outcomes include change in opioid dose and quality of life measures, and time to first rescue intravenous analgesic. Adverse events will be recorded.

**Discussion:**

NI-03 has been used successfully and safely in Japan to treat CP-associated pain. The aim of the current study is to assess the safety and efficacy of NI-03 using a rigorous RCT in a population in the USA. This study may fill an important clinical gap to provide an effective medical treatment option for CP-associated pain.

**Trial registration:**

ClinicalTrials.gov, NCT02693093. Registered through the National Institutes of Health on 26 February 2016.

**Electronic supplementary material:**

The online version of this article (10.1186/s13063-019-3606-y) contains supplementary material, which is available to authorized users.

## Background

Chronic pancreatitis (CP) is a progressive, fibro-inflammatory disease characterized by progressive inflammation resulting in loss of pancreatic endocrine and/or exocrine function [1]. In the absence of approved medical therapy to reverse or delay disease progression, management is focused on avoidance of known risk factors for disease progression (e.g., cigarette smoking and alcohol use) and monitoring for disease-related complications [2]. Unfortunately, a significant proportion of patients develop chronic abdominal pain during their disease course. This has been attributed to adjacent perineural inflammation and structural changes in the pancreatic parenchyma, including pancreatic ductal hypertension.

Surgical resection or drainage procedures and endoscopic therapy have been demonstrated to be effective for management of pain in the context of “large duct disease” (i.e., CP with main pancreatic duct obstruction), but effective therapies are needed for those without anatomic obstruction. While non-steroidal anti-inflammatory agents are commonly used in clinical practice, their efficacy has not been evaluated in human trials. A variety of medications have been examined in international studies including antioxidants, gabapentinoids, and serine protease inhibitors, with mixed results.

Vitamins with antioxidant properties are hypothesized to reduce the oxidative stress within the pancreatic microenvironment leading to improvement in CP-associated pain due to perineural inflammation [3]. Two large randomized controlled trials (RCTs) have been performed, demonstrating mixed results in efficacy [3–6]. Importantly, there were significant differences in the composition of the study cohorts, and antioxidants did not provide clear benefit in the cohort with a higher proportion of alcohol-induced CP [4]. Alternatively, pregabalin is a gabapentinoid that is hypothesized to reduce CP-associated pain through its effects on central processing [7]. In one RCT involving 64 subjects there was improvement in daily pain scores and quality of life [7]. However, the majority (91%) of subjects experienced a treatment-related adverse event related to treatment often requiring dose reduction (39%) or drug discontinuation (6%) [7]. Although promising, these results have not been externally validated.

Serine protease inhibitors, or anti-proteases, are hypothesized to inhibit pancreatic enzyme activity thereby reducing hyperamylasemia, inflammatory symptoms, and pain [8]. Camostat mesilate is a formulation that is widely used in Japan and is approved for treatment of acute symptoms of CP. Experimental evidence supporting the mechanism of action of protease inhibitors is largely derived from murine models of acute pancreatitis (AP). Treatment with protease inhibitors was shown to protect against pancreatic edema and hyperamylasemia in a cerulein-induced AP model [9]. Camostat mesilate reduced hyperamylasemia and mortality rate in a similar model involving a choline-deficient ethionine-supplemented (CDE) diet [9]. In both models, protease inhibitors reduced the migration of cathepsin B-containing auto-digestive enzymes to the periphery of the cell, a key step in the autolysis pathway of AP [9]. The inhibitory effect of camostat mesilate was dose-dependent in these studies, with higher doses achieving greater suppression of trypsin and kallikrein [10].

Several human studies have demonstrated the safety and efficacy of camostat mesilate, 200 mg three times daily, based on subjective complaints and objective findings in subjects treated for up to 8 weeks for AP and CP [11–21]. Importantly, the efficacy varied according to the etiology of pancreatitis and the severity of underlying symptoms, with improved responses in those with more severe CP [11, 12, 14, 15, 17, 20]. Subjects treated with a 600-mg total daily dose in a randomized, placebo-controlled trial in 287 subjects experienced “marked improvement,” which was defined on a 6-point scale (markedly improved, improved, slightly improved, unchanged, worsened, and markedly worsened) [14]. Several doses were used in these studies, ranging from a 300-mg to 900-mg total daily dose, with 600 mg in three divided doses being the most common [11–21]. The only study that included alternate dosing was not powered to determine a difference in efficacy among the different doses [20]. The approved dose in Japan is 600 mg by mouth in three divided doses [8]. It is important to note that these studies were conducted in Asian subjects and the pharmacokinetics and side effects may not be broadly applicable to other populations.

The effects of camostat mesilate on pancreatic function have also been examined. A small, double-blind RCT reported that short-term use of camostat mesilate improved maximal bicarbonate secretion, suggesting improved duct cell function compared to placebo [17]. This study did not distinguish between subjects with AP, confirmed CP, or suspected CP in their analysis of bicarbonate secretion [17]. In contrast, a larger double-blind RCT involving 287 subjects did not show improvement in either exocrine or endocrine pancreatic function among subjects with AP or CP after treatment for up to 6 weeks, so the impact on reversing functional abnormalities remains uncertain [14].

The reported safety profile of this class of drugs is favorable. Reported adverse effects are rare (< 3%) and typically mild (such as pruritus, increased thirst and appetite, and lightheadedness) [11, 14].

The primary objective of this study is to assess the safety and efficacy of NI-03, a serine protease inhibitor, for the treatment of CP-associated pain compared to placebo. In this trial we will initially evaluate the safety and pharmacokinetics after administration of a single dose of three different dosages of NI-03 to confirm acceptability of a dosage (300 mg) that is higher than previously studied. Subsequently, we will perform a double-blind, randomized, placebo-controlled trial of these three dosages of NI-03 to assess the efficacy for treatment of CP-associated pain.

## Methods

### Specific objectives

The objectives of the study are to:

1. Assess the pharmacokinetic and safety profiles following administration of a single dose of NI-03 (100 mg, 200 mg, and 300 mg).
2. Evaluate the efficacy and safety profile of NI-03 (100-mg, 200-mg, or 300-mg dosage) administered three times daily for 28 days for CP-associated pain in a double-blind, randomized, placebo-controlled trial.

### Study population

#### Inclusion criteria

The inclusion criteria are:

- Adults aged 18–85 years.
- Diagnosis of chronic pancreatitis supported by a combination of cross-sectional imaging, endoscopic ultrasound (EUS), endoscopic retrograde pancreatography (ERP), and/or assessment of pancreatic function (Tables 1 and 2) [22–24].
- Average baseline pain score ≥ 4/10 using a numeric rating scale (0–10) during the 7-day run in period.
- Stable analgesic regimen: if oral narcotic analgesics are utilized, the daily oral morphine equivalent (OME) dose should not exceed 100 mg.
- Ability to use contraception method from screening until 28 days after completion of the study medication.
- Ability to understand and provide written informed consent.

**Table 1:** Categories of supportive evidence for diagnosis of chronic pancreatitis in this study

Category	Description
1. Presence of pancreatic calcification(s)	Presence of one or more pancreatic parenchymal or ductal calcification(s) on cross-sectional abdominal imaging
2. Imaging supportive of chronic pancreatitis	Diagnosis requires the presence of a, b, or c: a) EUS demonstrating lobular appearance of the pancreas and ≥ 3 minor criteria (Table 2) b) EUS demonstrating ≥ 5 minor criteria c) Presence of ≥ 3 abnormal pancreatic duct side branches on MRCP or ERCP
3. Indeterminate EUS with evidence of exocrine pancreatic insufficiency	Diagnosis requires the presence of a and b, c, d, or e: a) Presence of 3–4 minor EUS criteria b) Abnormal 72-h fecal fat collection (> 15 g fat per day) c) Abnormal endoscopic pancreas function test (maximal duodenal bicarbonate concentration < 80 mEq/L) d) Decreased serum trypsin (< 20 ng/mL) e) Decreased fecal elastase level (< 200 mcg/g stool)

*EUS* endoscopic ultrasound, *MRCP* magnetic resonance cholangiopancreatography, *ERCP* endoscopic retrograde cholangiopancreatography

**Table 2:** Rosemont criteria utilized for diagnosis of chronic pancreatitis with endoscopic ultrasound (EUS) [22]

Major criteria	Minor criteria
• Hyperechoic foci with shadowing • Contiguous lobules with honeycombing	• Lobular appearance in pancreas • Hyperechoic foci without shadowing • Cysts • Hyperechoic stranding • Irregular main pancreatic duct contour • Dilated side branches • Main pancreatic duct dilation • Hyperechoic main pancreatic duct walls

#### Exclusion criteria

The exclusion criteria are:

- Comorbid medical conditions: including clinically significant cardiovascular disease, active infection within 30 days of day 1, seizure within the past 12 months, pregnancy or planned pregnancy or active breast feeding, history of malignancy within 5 years of study enrollment, or HIV infection.
- Renal or hepatic dysfunction: including stage IV chronic kidney disease (estimated using the Cockcroft-Gault formula). Active, chronic hepatitis B infection (surface antigen positivity), chronic hepatitis C infection (including a detectable PCR level or undetectable levels in advanced fibrosis (histologic grade 3–4/4), or cirrhosis based on previous evaluation including biopsy, a noninvasive estimate of fibrosis, or radiographic features.
- Diagnosis of autoimmune pancreatitis: based on the International Consensus Diagnostic Criteria for Autoimmune Pancreatitis [25].
- Use of potentially confounding medications: including other experimental medications, recent change in selective serotonin reuptake inhibitor (SSRI)/serotonin-norepinephrine reuptake inhibitor (SNRI) dosage, systemic steroids, anti-epileptics, or antipsychotics.
- Potential confounding of pain assessment: including the presence of generalized pain syndrome prohibiting the differentiation of abdominal pain, major abdominal surgery or endoscopic intervention (including celiac plexus block, sphincterotomy, and/or pancreatic duct stenting) within 90 days of enrollment.
- Substance abuse: including use of illegal substances, use of cannabinoids (subjects must have a 28-day wash-out period and negative drug test at screening and at day 29), or alcohol consumption exceeding 2 drinks per day (or 14 drinks per week).
- Miscellaneous: inadequate venous access, known hypersensitivity to NI-03 or one of its excipients, inability/unwillingness to comply with study restrictions, or blood donation or transfusion within 7 days of enrollment.

### Intervention

The study drug, NI-03, is camostat mesilate and will be manufactured in tablet form by Nichi Iko (Japan). Placebo tablets are manufactured by Stason Pharmaceuticals, Incorporated (Irvine, CA, USA). The drug will be placed into gelatin capsules in a manner that the tablet labeling is concealed and will be indistinguishable from placebo tablets. The encapsulated tablets will be distributed as a multiple dose card containing 24 capsules to account for three capsules per day and three extra capsules per week.

We will evaluate drug safety and pharmacokinetics during the single-dose study. We will recruit approximately 6–8 subjects per dose (100 mg, 200 mg or 300 mg NI-03) in this phase I study, giving a total of 18–24 subjects. After a single oral dose, blood samples will be drawn at 0.25, 0.5, 1, 2, 4, and 8 h. Samples will be collected, processed, and shipped according to standard operating procedures. Subjects will have a follow-up visit 1 week after the dose to evaluate subjects for adverse effects. These data will be submitted to the Food and Drug Administration (FDA) for formal safety evaluation before proceeding with further study.

**Fig. 1:**
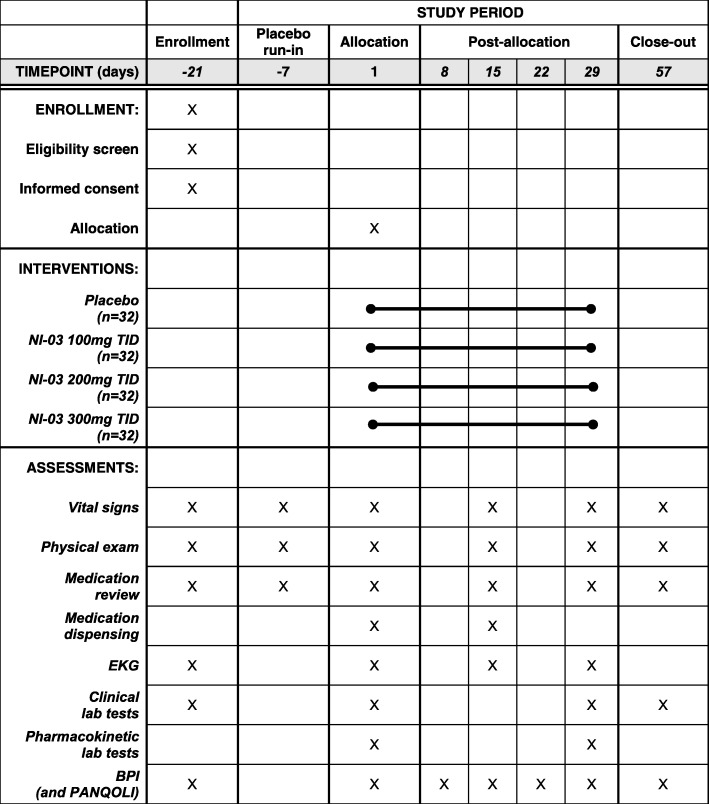
Standard protocol items: recommendation for interventional trials (SPIRIT) figure demonstrating the study timeline for the randomized, double-blind trial of NI-03 for pain control among subjects with painful chronic pancreatitis. BPI, Brief Pain Inventory; PANQOLI, Pancreatitis Quality of Life Instrument; TID, three times per day; EKG, electrocardiogram

The phase II study will begin with a 7-day, single-blind, placebo run-in period. During this week, baseline characteristics will be assessed and subjects will have the opportunity to become familiarized with the pain rating scales. After the 1-week run in, subjects with an eligible baseline pain score will be randomized to one of four arms (NI-03 at 300 mg, 200 mg, 100 mg, or placebo; all given three times daily) for a 28-day treatment period (Fig. 1). Subjects will have in-person study visits with blood draws on day 0 and day 29 for additional pharmacokinetic evaluation; blood for laboratory tests will be drawn prior to administration of the dose and 0.25, 0.5, 1, 2, and 4 h post-dose. An additional visit will take place in person on day 15 for completion of study assessments including review of dosing cards. Subjects will be contacted by telephone on day 8 and day 22 to assess them for adverse effects. An end of study visit will take place on day 57.

### Randomization and blinding

A randomization scheme was developed for each site based on participants’ baseline OME dosage (i.e., “Low” (0–50 mg/day) versus “High” (50–100 mg/day)). Subjects will be randomized by block. Each site has 6 reserved blocks, including 4 blocks of 4 units and 2 blocks of 8 units (representing a total of 32 units at each site). Following randomization, investigators and participants will be blinded to treatment assignments for the remainder of the study.

### Outcomes and outcome measures

The primary outcome is change in average daily worst pain intensity score. Daily pain intensity will be scored using a numerical rating scale from 0 to 10. These values will be averaged over each week of the study, with the primary comparison between the placebo run-in period and the terminal 7 days of the treatment phase (i.e., days 22–28). Averaging pain scores over 1 week will adjust for those subjects with intermittent pain. Those with infrequent pain will be excluded if the average weekly pain score is < 4/10.

Secondary outcomes include change from baseline in least pain score, average pain score, current pain score, change in average OME and gabapentin/pregabalin daily dosages, time to first intravenous analgesic use (as rescue therapy), and change in quality of life assessed by the Pancreatic Quality of Life Instrument (PANQOLI) [26] and pain interference aspects of the brief pain inventory [27] compared to baseline. Time to first intravenous analgesic will be measured from day 1 of the trial to use of any intravenous analgesic.

### Study recruitment and sample size justification

Study recruitment will occur at 45 centers in the USA. Various methods will be used to identify potential participants, including surveillance of clinic schedules, notification of potential referring providers, and direct advertisement to potential participants using research match tools. Trained study personnel at each site will recruit subjects.

Assuming a one-unit difference between each NI-03 dose and placebo, and a standard deviation (SD) of 1.6, the initial total sample size is calculated as 120 subjects. This will yield approximately 80% power at an overall two-sided type 1 error rate of 5% using Dunnett’s procedure. Assuming up to 5% of subjects will not qualify for the full analysis set due to incomplete data collection or failure to follow up, the total initial sample size is increased to 128 subjects. When approximately 50% of the subjects have completed the phase II study, an interim analysis will be performed to update the SD estimate primary endpoint, and the sample size will be recalculated. Data quality will also be reviewed during the interim analysis.

### Data and statistical analysis

All subjects who receive at least one dose of NI-03 and have baseline and post-baseline pain scores will be included in the dataset for analysis. Subjects’ data will be analyzed according to their assigned treatment (i.e., intention to treat). An additional per-protocol analysis is planned to evaluate only the data from subjects without protocol deviations (Additional file 1).

The primary efficacy variable, change in average daily worst pain score, will be analyzed using a restricted maximum likelihood (REML)-based repeated measures approach (i.e., a mixed model repeated measures (MMRM) analysis). The MMRM model will include treatment, stratification factor (oral morphine equivalent dose 0–50 mg/day versus 50–100 mg/day), center, baseline average daily pain intensity score, visit, and treatment by visit interaction as fixed-effect explanatory variables. The study center will be included in the model as a random effect. Additionally, analysis of covariance (ANCOVA) of the change from baseline to week 4 in the average daily worst pain intensity score, with the baseline score as the covariate, both treatment and stratification factor as fixed effects, and center as a random effect, will be assessed in a robustness analysis using the baseline observation carried forward (BOCF) approach.

Regular monitoring of study sites will be performed by an independent contract research organization (CRO) throughout the study period with a focus on processes for handling, reporting, and archiving data. An additional data monitoring committee will not be used for this study as the CRO will be involved with auditing study procedures and completeness of case report forms. Data sharing among study investigators will occur via secure email. The final trial dataset will be available to the Sponsor and co-authors of the study manuscript.

### Patient safety and adverse event recording

Safety will be monitored through weekly contact: telephone calls during weeks 1 and 3 and office visits during weeks 2 and 4. All adverse events will be recorded. The investigator will determine both the severity of the event and relationship to study treatment, based on clinical judgment. Subjects may discontinue treatment if adverse events occur or they may be removed from treatment as determined by the investigator. Rationale for drug discontinuation will be reported. The investigator will not be un-blinded if a serious adverse event occurs. Every serious adverse event (death, life-threatening event, hospitalization, persistent disability, or other important medical event) will be reported to the lead investigator as soon as possible to determine causality. The event will be submitted expeditiously in the event of an adverse drug reaction. Medical care for study-related adverse events will be paid for by the study Sponsor.

## Discussion

This phase I/II study will assess the safety and efficacy of NI-03 for the treatment of CP-associated pain in a double-blind, randomized, placebo-controlled trial. During the phase I study, a pharmacokinetic evaluation will occur after one dose, with safety assessments at 1 week. The subsequent phase II study will consist of a 1-week, single-blind, run-in period followed by a 4-week, double-blind treatment period to compare the change in average daily worst pain intensity scores. Key secondary outcomes will include changes in concurrent analgesic dose and quality of life scores. The purpose of this study is to fill an important clinical gap in providing an effective medical treatment option for CP-associated pain.

The management of painful CP begins with abstinence from alcohol and tobacco and often includes assessment for local complications or pancreatic duct obstruction, which may lead to procedural or surgical interventions. Analgesia is commonly provided in keeping with the World Health Organization pain ladder for those in whom anatomic intervention is not clearly warranted. Unfortunately, these interventions involve generic management of chronic pain and do not target the underlying mechanisms leading to CP-associated pain or CP-related disease progression.

Oral analgesic agents, including antioxidants and pregabalin, addressing the mechanisms of painful CP have been previously studied [3–7]. However, antioxidants have not been consistently shown to improve pain scores in patients with CP [3–6]. Pregabalin has demonstrated improvement in a small trial, but its use was limited due to side effects [7]. Serine protease inhibitors, including an oral agent called camostat mesilate or NI-03 have been approved for use in Japan to treat painful CP [8]. However, the original studies were conducted primarily on male patients of Asian descent during the late 1970s, and their results may not be applicable to an American demographic including women and other ethnicities. Furthermore, the original studies did not evaluate safety or efficacy of doses other than 200 mg three times daily. Therefore, a modern study including an evaluation of the pharmacokinetics of varying doses is required. Additionally, subjects need to be randomized to varying doses to determine the optimal dose for safety and efficacy.

Pain remains a common and frequently debilitating symptom for many patients with CP. Despite the associated morbidity, high-quality evidence-based treatment options remain limited. This study will evaluate the pharmacokinetics, safety, and efficacy of an orally available serine protease inhibitor, NI-03, for CP-associated pain. If efficacious, this will be a novel agent for treatment of symptoms among patients with CP in the USA.

## Trial status

This trial was prospectively registered through the National Institutes of Health on 26 February 2016 (NCT02693093). Enrollment for the phase I study began in December 2015. Enrollment for the phase II study began in April 2017 and remains ongoing. Any amendments to the above protocol will be reported with the study publication.

## Additional file

Additional file 1: Standard protocol items: recommendation for interventional trials (SPIRIT) checklist for the current study. (DOC 121 kb)

## Data Availability

Not applicable. Upon completion of the trial, data will be made available according to the requirements for all registered clinical trials at ClinicalTrials.gov.

## Abbreviations

AP: Acute pancreatitis
CP: Chronic pancreatitis
EUS: Endoscopic ultrasound
OME: Oral morphine equivalent
PK: Pharmacokinetics
RCT: Randomized controlled trial
